# Effects of self-management interventions in adults with acute coronary syndrome: A systematic review and meta-analysis of randomized controlled trials

**DOI:** 10.15446/rsap.V25n2.106585

**Published:** 2023-03-01

**Authors:** Roxana De las Salas, Lorena Isabel Velasco-Banquet, Elizabeth Villarreal-Cantillo, Marta Palmet-Jiménez

**Affiliations:** 1 RD: Registered Nurse. M.Sc. Sciences Pharmacology. Ph.D. Pharmaceutical Sciences. Assisstant Professor, Department of Nursing. Universidad del Norte. Barranquilla, Colombia. rdelassalas@uninorte.edu.co Universidad del Norte Department of Nursing Universidad del Norte Barranquilla Colombia rdelassalas@uninorte.edu.co; 3 LV: Registered Nurse. M.Sc. Nursing Sciences. Department of Nursing. Universidad del Norte, Barranquilla, Colombia. lbanquet@uninorte.edu.co Universidad del Norte Nursing Sciences Department of Nursing Universidad del Norte Barranquilla Colombia lbanquet@uninorte.edu.co; 4 EV: Registered Nurse. M.Sc. Education. Emeritus Professor, Department of Nursing, Universidad del Norte. Barranquilla, Colombia. evillare@uninorte.edu.co Universidad del Norte Department of Nursing Universidad del Norte Barranquilla Colombia evillare@uninorte.edu.co; 5 MP: Registered Nurse. M.Sc. Nursing Sciences. Department of Nursing, Universidad del Norte. Barranquilla, Colombia. mpalmet@uninorte.edu.co Universidad del Norte Nursing Sciences Department of Nursing Universidad del Norte Barranquilla Colombia mpalmet@uninorte.edu.co

**Keywords:** Self-management, nursing, acute coronary syndrome, evidence-based nursing, meta-analysis, systematic review *(source: MeSH, NLM)*, Automanejo, enfermería, síndrome coronario agudo, enfermería basada en la evidencia, metaanálisis, revisión sistemática *(fuente: DeCS, BIREME)*

## Abstract

**Objective:**

The aim of this study is to determine the effects of self-management interventions in adults with Acute Coronary Syndrome.

**Methods:**

Systematic review and meta-analysis. The Prisma checklist was used. Embase via Ovid, Lilacs, Medline via PubMed and Central were searched for randomized controlled trials. The Cochrane collaboration guidelines were followed and reported using the Preferred Reporting Items for Systematic Reviews and MetaAnalyses (Prisma) statement. The Revman program was used to consolidate the data. A sensitivity analysis was carried out.

**Results:**

A total of 17 randomized controlled trials met eligibility criteria. The results showed that self-management interventions did not significantly enhance patients' compliance with medication OR=1.32 (95% CI 0.66-2.64), lifestyle changes OR=1.08 (95% CI 0.55-2.15) and modification of clinical variables MD=-1.77 (95% CI -2.96 6.50).

**Conclusion:**

This review suggests that there are no statistically significant differences between self-management interventions and the usual care given to patients with Acute Coronary Syndrome Compliance with Pharmacological Treatment, lifestyle changes and Changes in Clinical Variables.

## RESUMEN

**Objetivo:**

El objetivo de este estudio es determinar los efectos de las intervenciones de automanejo en adultos con síndrome coronario agudo.

**Métodos:**

Revisión sistemática y metaanálisis. Se utilizó la lista de verificación Prisma. Se realizaron búsquedas de ensayos controlados aleatorizados en Embase, mediante Ovid, Lilacs, Medline por medio de PubMed y Central. Se siguieron las guías de colaboración Cochrane y se informaron utilizando la declaración *Preferred Reporting Items for Systematic Reviews and MetaAnalyses* (Prisma). Se utilizó el programa Revman para consolidar los datos. Se realizó un análisis de sensibilidad.

**Resultados:**

Un total de 17 ensayos controlados aleatorizados cumplieron con los criterios de elegibilidad. Los resultados mostraron que las intervenciones de automanejo no mejoraron significativamente el cumplimiento de la medicación por parte de los pacientes OR= 1,32 (IC 95% 0,66-2,64), los cambios en el estilo de vida OR=1,08 (IC 95% 0,55-2,15) y la modificación de las variables clínicas MD=-1,77 (IC 95% -2,96-6,50).

**Conclusión:**

Esta revisión sugiere que no existen diferencias estadísticamente significativas entre las intervenciones de automanejo y la atención habitual que se brinda a los pacientes con síndrome coronario agudo, cumplimiento del tratamiento farmacológico, cambios en el estilo de vida y cambios en las variables clínicas.

According to the World Health Organization (WHO), cardiovascular diseases (CVD) are the leading cause of death and disability worldwide. In 2019, WHO reported 17.9 million deaths due to cardiovascular diseases, which represented 32% of all global deaths 1.

Most cardiovascular diseases can be prevented by intervening in behavioral risk factors such as tobacco use, unhealthy diets, obesity, physical inactivity, and harmful alcohol use 1. According to the American Heart Association, the control of risk factors in adults by interventions has led to a decrease in mortality from myocardial infarction 2.

Several studies have shown that interventions aimed at reducing the recurrence of Acute Coronary Syndrome (ACS), with combined interventions among them, such as educational and website-use based interventions, that promote adherence to prevention and lifestyle changes and favor self-management and healthy outcomes in patients with this disease 3-5.

Although the concept of self-management has been discussed in various contexts, the WHO proposes that the cornerstone for health care for human beings with chronic diseases was precisely self-management 6. With this, it seeks to use effective nursing interventions, supported by scientific evidence, in order to improve the quality of life in this population 7.

It is essential to provide nurses and clinicians with strong evidence that the interventions of self-management improve in enhancing patients' Compliance with Pharmacological Treatment, Changes in Lifestyle and in Clinical Variables. There have been some meta-analyses published reporting on the effect of self-management on several clinical conditions and among various populations 8-10, but still, it is not yet clearly understood the effects of self-management in patients with ACS. Hence, the findings of this meta-analysis will provide evidence regarding the best Self-Management Interventions to the health professionals in creating healthcare policies worldwide.

## METHODS

### Aim

A systematic review and meta-analysis were performed to determine the efficacy of interventions for self-management in adults with Acute Coronary Syndrome.

### Design

The present systematic review and meta-analysis was carried out according to the standard protocol of the Cochrane Handbook for systematic reviews and was based on the Preferred Reporting Items for Systematic Reviews and Meta-Analyses (Prisma) 11. This study has been registered in the International Platform of Registered Systematic Review and Meta-analysis Protocols (INPLASY) (#202260045).

### Search methods

A search was carried out in the electronic databases Embase via Ovid, Lilacs, and Medline via PubMed and Central for randomized controlled clinical trials, in English and Spanish, published by June 2020. Based on PICO format (population, intervention, control and outcomes), search strategy was formed using relevant key terms, and two authors independently searched the databases for randomized controlled trials (RCTS). The terms used were adjusted for each of the databases using Mesh and Decs Bireme (Table 1). The inclusion criteria were as follows.

**Table 1:** Database search strategy

Database	Search strategy
Medline via Pubmed	((("Acute Coronary Syndrome"[Mesh]) OR "Coronary Disease"[Mesh]) AND "Self-Management"[Mesh]) OR "Self Efficacy"[Mesh]
Embase via Ovid	('acute coronary syndrome')/exp OR (('heart muscle ischemia')/de) AND ((self-management)/exp/mj) OR (('self-concept')/de)
Lilacs	Acute Coronary Syndrome [DeCS Category] or Coronary Disease [DeCS Category] and Self-Management [DeCS Category] or Self Efficacy [DeCS Category]
Central	"acute coronary syndrome" in Title Abstract Keyword OR "coronary disease" in Title Abstract Keyword AND "self-management program" in Title Abstract Keyword OR "self efficacy" in Title Abstract Keyword

#### Participants

We focused on Adults with Acute Coronary Syndrome. 

#### Intervention

Patients must have received Self-management interventions. The studies evaluated the effects of self-management on Compliance with Pharmacological Treatment, Changes in Lifestyle and Changes in Clinical Variables. It was defined as Long-term follow up ≥ 6 months and Short-term follow-up as <6 months.

#### Control

The control group included Standard Care for patients. The patients randomized to standard care follow the usual primary or secondary prevention practices of the center to which they had been admitted.

#### Outcomes

Effects of self-management on Compliance with Pharmacological Treatment, Changes in Lifestyle and Changes in Clinical Variables were specific measures that were considered for inclusion. In the studies, results that would allow for the evaluation of the efficacy of self-management interventions were sought. These related to the following outcomes: Compliance with Pharmacological Treatment, made up of two subgroups, adherence to medication and achievement of treatment; Changes in Lifestyle and Changes in Clinical Variables, with five subgroups, maintenance of physical exercise, quitting smoking, increased consumption of vegetables and fruits, and reduction of alcohol consumption; and the effect on clinical variables, made up of five subgroups: weight, body mass index, cholesterol, systolic blood pressure, and HgbAic.

The patients randomized to standard care follow the usual primary or secondary prevention practices of the center to which they have been admitted. The trials that included samples with less than 18 years old, published in languages other than English, did not include self-management on Acute Coronary Syndrome, and non-RCTS; were excluded from this review.

### Data abstraction

Two independent reviewers (RD and LV) screened manuscript titles and abstracts to identify potentially relevant studies describing self-management interventions in adults with Acute Coronary Syndrome. Disagreements between the two reviewers were resolved by discussion with a third reviewer (EV). Articles with high eligibility were evaluated in full text. The bibliography of the identified studies was also hand searched. Duplicate manuscripts were removed after transferring the search results to EndNote® Web (Clarivate Analytics, Philadelphia, PA, USA).

### Quality appraisal

Eligible studies were independently assessed for risk of bias using the Cochrane Risk of Bias (ROB) tool 12. In addition, the level of evidence was classified according to the Oxford Center for Evidence-Based Medicine tool. The five domains of risk of bias.

### Data analysis

The primary analysis involved comparisons of the effect of self-management interventions on the aforementioned outcomes. For dichotomous variables, Odd Ratio (OR) was estimated with its 95% confidence interval (CI). For continuous variables, the Mean Difference (MD) with its 95% CI was used. In addition, statistical heterogeneity 12 was assessed. All of the above, using Review Manager (Revman) version 5.4.1.

The meta-analysis included a subgroup analysis of results, an assessment of statistical heterogeneity, by groups and in general, was included. In addition, the visual assessment of the meta-analytic model was performed by inspecting the forest plot for each result. Further, sensitivity analysis using the 'Exclude results from specific studies' method was conducted to assess each study's impact on the combined effect.

## RESULTS

### Search strategy

The search strategy produced 3 403 potentially relevant publications (Table 2).

**Table 2:** Interventions for Self-Management in Adults with Acute Coronary Syndrome (ACS): Systematic Review of the Literature

c	Founding source	Study population (country)	Type of study Sample	Main Intervention (Intervention / Control)	Duration	Result	Oxford level of evidence	Oxford level of evidence
TIMLI, 2002	The Minnesota Agricultural Experiment Station Publication (Project MIN-54-026).	Men and women, 35 to 85 years old (USA)	Controlled trial	130	Nutritional education by a multidisciplinary group led by a dietician. Intervention group: attended two group nutrition education classes and one individual dietary counseling session. Control group: received regular non-individualized nutritional education from cardiac rehabilitation therapists.	6 weeks plus a 3 months follow-up.	The treatment group had a greater improvement in Restaurant and Recipe scores on the Diet Habits Survey (2.6 vs 1.0), and a greater mean self-efficacy score on the cardiac diet (4.3), compared to the control group (3.8). The saturated fat cholesterol index decreased significantly in the control group (from 57 to 48), and in the treatment group (from 51 to 42).	1a
Kukafka, 2002	The National Library of Medicine	Men and women, average age of 57. (USA)	Randomized controlled trial	94	Benefits of using web pages (tailored messages) The participants were assigned to one of three groups: (1) personalized web-based, (2) non-personalized web-based, and (3) non-personalized paper-based.	3 months	A trend toward improvement in self-efficacy scores was found in all groups at the one-month mark follow-u p, with significant and sustained increases in baseline scores at 3 months only in the personalized web-based group.	1a
Scott, 2004	The Robert Wood Johnson Chronic Care Initiative	Men and women ≥60 years old (USA)	Randomized controlled trial	254	Adherence to prevention Intervention: Monthly group meetings held by patients’ primary care physicians and a nurse every month for 90 minutes. Control: patients continued to receive the same care from their primary care physician that they had received before.	24 months	The model resulted in fewer hospitalizations and emergency visits, higher patient satisfaction and self-efficacy, but no effect on outpatient use, health, or functional status.	1b
Wiggers, 2005	The Netherlands Heart Foundation	Men and women, average age of 59 (Netherlands)	Randomized controlled trial	315	Cognitive and educational intervention of lifestyle change for self-efficacy and the intention to quit smoking. Intervention: Minimal Intervention Strategy for Cardiology patients (C-MIS) Control: Nicotine Replacement Therapy (NRT) or NRT + C-MIS.	12 months	C-MIS did not significantly influence the decision to quit smoking. However, effects of the C-MIS on the intention to quit smoking and self-efficacy in patients with higher education were found.	1a
Izawa, 2005	The Japanese Society for the Promotion of Science	Men and women, average age of 64,2 (Japan)	Randomized controlled trial	45	Adherence to prevention. Intervention: self-monitoring approach on self-efficacy for physical activity (SEPA), exercise maintenance, and objective physical activity level over a 6-months period after a supervised 6-months cardiac rehabilitation (CR) program. Control: participated in routine care.	6 months	The mean SEPA (self-efficacy for physical activity) score (90.5 vs 72.7 points, p 0.001) and mean objective physical activity (10.458.7 vs 6922.5 steps / week, p 0.001) at 12 months after the onset of myocardial infarction.	1a
Shively, 2005	The Department of Veterans Affairs, Veterans Health Administration, Health Services Research and Development Service	Men and women, 41 to 90 years old, average age of 67 (USA)	Randomized controlled trial	116	Educational intervention to change lifestyles (nurse-led behavior management). Intervention: behavioral management program consisted of four classes (2h each one) and three phone calls over a 4-month period. Control: usual care for patients with heart Failure.	16 months	Intervention patients showed a significant improvement in disease-specific health-related quality of life over time. There were no group differences in exercise performance, physical and others.	1a
Carroll, 2006	The National Institute of Nursing Research and the Charles Farnsworth Trust.	Older adults of 65 years of age (USA)	Randomized controlled trial	279	Educational intervention to change lifestyles Interventions: Peer advisor and an advanced practice nurse (APN). Control: standard care.	12 weeks	There were no significant differences between the 3 groups in health outcomes.	1a
Muñiz, 2010	Merck, Sharp & Dohme and University of Coruña.	Men and women, 18 to 80 years old, after an episode of Acute Coronary Syndrome (ACS) (Spain)	Randomized controlled trial	1510	Educational intervention on modifiable risk factors and pharmacological compliance six months after an ACS. Intervention: personalized interview at discharge. Control: usual care given.	6 weeks	Differences were observed in the mean reduction of body mass index (0.5 vs. 0.2; p <0.001) and waist circumference (1.6 cm vs. 0.6 cm; p = 0.05), those with total cholesterol below 175 mg / dl (64.7% vs. 56.5%; p = 0.001), with reduction of hospitalizations and mortality.	1b
Furze, 2012	British Heart Foundation	Men and women with a mean age of 64 and a diagnosis of angina. (United Kingdom)	Randomized controlled trial	142	Educational program Intervention: home angina management program. Control: advice from an angina nurse specialist.	6 months	There were no important differences in the frequency of angina at 6 months. The secondary outcomes showed differences in favor of the intervention group.	1b
Sheridan, 2013	The U.S. Centers for Disease Control and Prevention, American Recovery and Reinvestment and National Institutes of Health	Men and women from 35 to 79 years old, mean age 62.3 years (USA)	Randomized trial	489	Web-based pharmaceutical intervention of a combined intervention on lifestyle and medication adherence. Intervention: provided by a counselor Control: web-based alternative intervention.	12 months	Adherence to medication (secondary result) to better control of blood pressure. They verified high adherence by correlating it with the expected changes in blood pressure (-4 mm Hg) and cholesterol (-20 mg / dl) based on 50% of the standard change reported in meta-analysis of treatment trials.	1a
Hawkes, 2012	National Health and Medical Research Council and National Heart Foundation of Australia	Men and women aged 18 to 80 years, diagnosis of MI. Mean = 60.6 years. (Australia)	Randomized, prospective and parallel group controlled trial	430	Secondary prevention program Intervention: Health Coaching Control: Usual care.	6 months	Significant effects for healthrelated quality of life were observed on the mental component summary score and the social and emotional functioning subscale in intervention group.	1a
Devi, 2014	The National Institute for Health Research (NIHR), Collaboration for Leadership in Applied Health Research and Care East Midlands	Men and women, with an average age of 66.2 years (United Kingdom)	Randomized controlled trial with 2 arms of parallel groups	94	Web-based cardiac rehabilitation program for people with angina. Intervention: delivered via the Internet and called “Activate Your Heart” designed for participants to use at home. emotions. Control: treatment as usual from their GP and received no further contact from the researcher until the 6-week follow-up.	6 months	Daily physical activity improved according to step count (the main outcome). There are also significant improvements in secondary outcomes such as reduced sedentary time and increased time spent being moderately active.	1a
Zhao, 2015	None	Men and women over 18 years of age (China)	Controlled clinical trial	90	Pharmaceutical intervention Intervention: conventional medical treatment plus interventions by clinical pharmacists who developed individual drug regimens based on each patient’s needs and condition. Control: conventional medical treatment without pharmacist participation.	6 months	Pharmaceutical support improved self-care capacity, quality of life, and compliance with treatment in patients with coronary artery disease.	1a
Duan, 2018	Hong Kong Baptist University	Men and women aged between 20 to 75 years, with a mean = 49.18 (China)	Randomized controlled trial	136	Web based intervention Intervention: The intervention content was designed based on the HAPA theory. Control: waiting control group.	8 weeks	The psychological resources of patients, such as motivation, self-efficacy, planning and social support, as well as lifestyle, can be improved through a web-based intervention.	1a
Paoli, 2018	Emilia- Romagna’s regional government and AstraZeneca.	Patients> 18 years of age with ACS (unstable angina) (Italy)	Prospective, randomized, multicenter, and interventional	2060	Educational intervention Intervention: Intensive secondary prevention program in which each patient goes to 9 one-to-one educational sessions with a trained nurse: one before discharge and the others after 1,3,6,12,18,24,36, and 48 months. Control: standard care.	5 years	The primary endpoint was achievement of goals related to risk factors, lifestyle modifications, and drug adherence after 2 years of follow-up.	1a
Fernandes, 2020	None	Men and women, mean age 63.78 years. (Portugal)	Randomized, controlled clinical study	121	Hospital psychoeducational intervention Intervention: psychoeducational intervention on knowledge about ACS, control of risk factors, and adaptive health habits and lifestyle. Control: Standard care.	2 months	The hospital psychoeducational intervention had a positive effect on the knowledge about ACS, the control of risk factors and the promotion of positive health habits, and is effective in improve cardiac rehabilitation.	1a
Jiang, 2020	The National Social Science Fund of China	Men and women, over 18 years of age with an average age of 61.01 years, (China)	Randomized controlled clinical trial	144	Multidisciplinary self-management educational program led by nurses for patients with coronary artery disease. Intervention: A program that consisted of individual assessment, group health education, individual consultation, and follow-up. Control: routine care provided by the community health care center. Routine care: health information consultation and telephone follow-up services every 2 months. Telephone follow-up focused on general health inquiries and unstructured social chatting.	6 months	The multidisciplinary nurse-led self-management program resulted in a significant improvement in self-management behaviors, self-efficacy, and health-related quality of life, as well as a reduction in unplanned use of health services among patients with coronary heart disease in Chinese	1a

After examining titles and abstracts, 273 full-text publications with a high probability of inclusion were evaluated.

**Figure 1:**
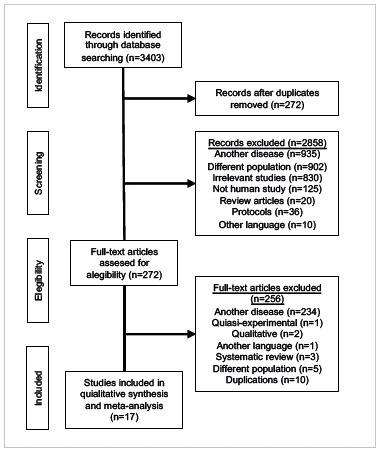
PRISMA flow diagram of study selection

Of these, 17 met the eligibility criteria, and were thus included in the descriptive synthesis (Figure 1).

### Descriptions of included studies

All studies included a total of 6 423 participants (range 45-2 060) and with a mean age of 61.49 years. Out of the 17 included studies, eight developed educational interventions 4,5,13-18; four, web-based interventions 19-22; three, interventions related to adherence to prevention 23-25; one, psychoeducational intervention 3; one, pharmaceutical intervention 26. Fifteen of the studies included men and women, the other two did not report information on sex. The studies were carried out in the United States (n = 6), the Netherlands (n = 1), Japan (n=1), Spain (n=1), the United Kingdom (n=2), Australia (n=1), China (n=3), Italy (n=1), and Portugal (n=1).

### Summary of ROB

The potential risk of bias was assessed using the seven domains for all included studies. In general, the selected articles met the established criteria, since most of them had a low risk of bias in the evaluation. Only one study was found to have a high risk of performance bias 5. Four studies had a high risk of attrition bias 3,5,20,22. Three of the studies showed a high risk of detection bias 3,5,20. While only one study demonstrated high risk of selection bias 13. Performance bias was largely affected by incomplete outcome data and blinded participants and staff.

### Pooled analysis: Health outcomes of self-management interventions

#### Compliance with Pharmacological Treatment

**Figure 2:**
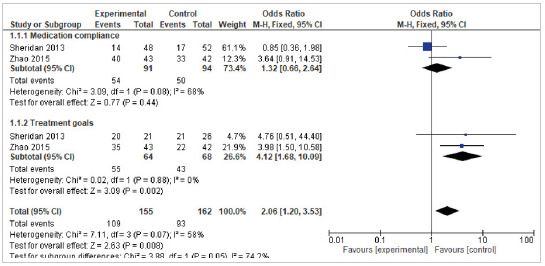
Effect of self-management interventions on compliance with pharmacological treatments (Long-term follow up > 6 months)

The first subgroup analysis included two studies that evaluated medication compliance and they showed an OR of 1.32 (95% CI 0.66-2.64), with a heterogeneity of 68%; for the treatment goals subgroup, an OR of 4.12 (95% CI 1.68-10.09) and a heterogeneity of 0%. The meta-analysis findings for this outcome indicate an OR of 2.06 (95% CI 1.20-3.53) and a heterogeneity of 74.2%, that is, the results are significant in favor of the control group (Figure 2).

#### Changes in Lifestyle

**Figure 3:**
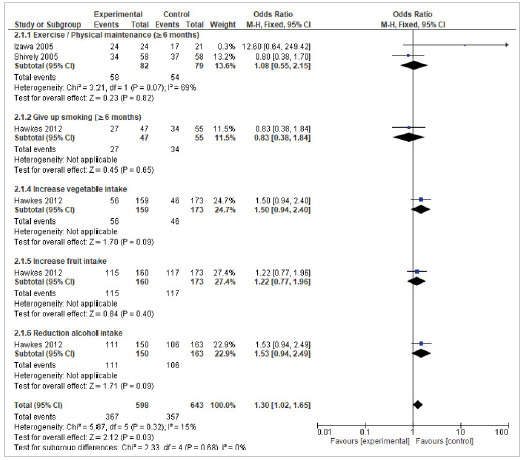
Effect of self-management interventions on changes in lifestyle (Long-term follow up ≥ 6 months).

The subgroup analysis for maintenance of physical activity included two studies with an OR of 1.08 (95% CI 0.55-2.15) and a heterogeneity of 69%. Other evaluated subgroups, that included a single study, were smoking cessation (OR=0.83 95% CI 0.38-1.87), increased consumption of vegetables (OR=1.50 95% CI 0.94-2.40), fruits (OR = 1.22 95% CI 0.77-1.96), and reduction of alcohol consumption (OR = 1.53 95% CI 0.94-2.49). According to the findings of the meta-analysis for this result, an OR of 1.30 (95% CI 1.02-1.65) and a heterogeneity of 0% were obtained, that is, the results are significantly in favor of the control group (Figure 3).

#### Changes in Clinical Variables

**Figure 4:**
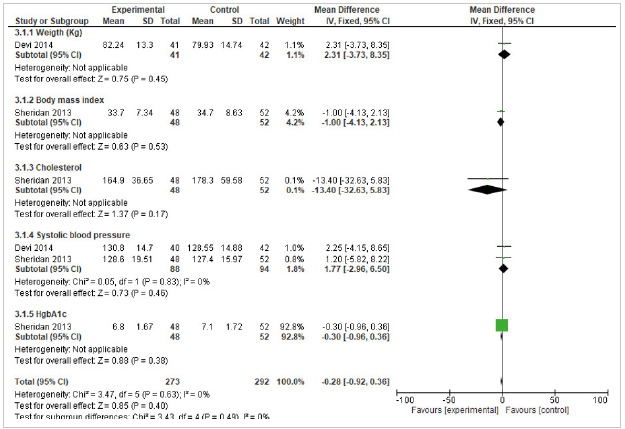
Effect of self-management interventions on clinical variables (Long-term follow up ≥ 6 months).

The subgroup analysis for the systolic blood pressure included two studies with a mean difference of -1.77 (95% CI -2.96 6.50) and a heterogeneity of 0%. Other evaluated subgroups, that included a single study, were weight control (mean difference 2.31 95% CI -3.73 8.35), body mass index (mean difference -1.00 95% CI -4.13 2,13), cholesterol (mean difference -13.40 95% CI -32.96 5,86) and glycosylated hemoglobin (mean difference -0.30 95% CI -0.96 0,36). According to the findings of the meta-analysis for this result, a mean difference of -0.28 (CI95% -0.92 0.36) and a heterogeneity of 0% were found, that is, the results are significant in favor of self-management interventions (Figure 4).

### Sensitivity analysis

The forest plot displayed no evident outlier for outcomes. This means that it is probable that one study alone could not substantially skew or drive the Odd Ratio in either one of the directions. When short term follow-up (<6 months) studies were added, the results did not change significantly for Compliance with Pharmacological Treatment and changes in lifestyle. Only for modification of clinical variables, the results were significant in favor of self-management interventions when short term follow-up (<6 months) studies were added.

## DISCUSSION

This systematic review and meta-analysis evaluated the effect of self-management interventions in adults with Acute Coronary Syndrome. Due to the increase in chronic non-communicable diseases, the rates of acute coronary syndrome and coronary heart disease have increased as well as complications and the poor control of risk factors (2), due to this, in the framework of clinical research, the possible benefits of programs that support the self-management of coronary disease have been established in order to reduce its effects on morbidity and mortality. However, the included trials did not assess mortality, reinfarction rates, rehospitalizations, or emergency room visits. The evaluated results focused on evaluating the efficacy of the interventions in relation to medication compliance, lifestyle changes, and the modification of clinical variables that are considered as intermediate results.

According to the evidence of the present meta-analysis, the impact of self-management programs did not show a significant change compared to conventional follow-up programs in adults with acute coronary syndrome for the results in Compliance with Pharmacological Treatment, lifestyle changes and Changes in Clinical Variables. This finding is comparable with other studies 8-10.

To achieve healthy behaviors, the family must be involved 27, that is, interventions should not focus only on individuals, but also on their families and their environment. Likewise, in their advancement, self-management processes require the perspective of people living with chronic diseases, taking into consideration three categories of self-management processes such as "focus on the needs of the disease", "activation of resources", and "living with a chronic disease" 28.

That is, self-management oriented towards how to recognize and control physical symptoms (pain, dyspnea, fatigue) and emotions (uncertainty, stress, anger, depression, and anxiety), in addition to managing daily life situations related to the chronic condition. While some authors 16,25 use the SF -36 instrument to establish measurements on physical and mental health, others 4 use the SF-12 and the MacNew Emotional Score 22. The foregoing indicates that there is a variability in the used assessment tools, which limited the comparison with this result.

In the included studies, interventions related to lifestyle changes were mostly led by nurses. The subgroup analysis for maintenance of physical activity had a heterogeneity of 57%, this can be partially explained by the different strategies used to motivate physical activity. In clinical trials, there is a greater concern for increasing and maintaining the level of motivation, however, it is shown that successful lifestyle interventions are related to the use of techniques to work on behavior as well as group and individual support 29.

On the other hand, the interventions aimed at quit-ting smoking were only significant for people with a high educational level 14. This, with the application of an intervention based on the behavioral theory The Minimal Intervention Strategy for Cardiology patients (C-MIS), which measures the disposition to change. One investigation proposed the reduction of cigarette consumption from an educational intervention 18 and, while another reported that patients were referred to the smoking cessation service 17, it does not expand on the details of said intervention, while Hawkes et al. mention the realization of consultancies 25. Although in the subgroup analysis a good consistency of the studies was obtained, the results indicate that there is no significance in relation to self-management interventions to quitting smoking, the vast majority of participants report difficulties in achieving this, which are largely linked to the addictive properties of nicotine 30. Another study 31 shows the influence attributed to the different reasons for quitting smoking, such as physical problems, the personal feeling of dependence, and health advice.

Regarding the consumption of vegetables and fruits, Hawkes et al. mention telephone health counseling sessions with a guideline script from the Australian National Heart Foundation, in collaboration with a health advisor for dietary intake. A web-based intervention, with the Health Action Process Approach (HAPA) as a theoretical context, suggests that there are 2 distinctive phases during the health behavior change process 20.

Regarding the modification of physical and clinical variables, the promotion of daily exercise based on website showed a positive impact on weight reduction, systolic blood pressure, and angina symptoms at six months. Social quality of life scores increased for the intervention group 22.

Adherence to medication was measured using the Morisky score, correlating high adherence with changes in blood pressure, cholesterol, and glycosylated hemoglobin, among others. The foregoing, in order to reduce the risk of acute coronary syndrome and with very favorable results for the intervention group. According to the findings of the meta-analysis for the subgroup of changes on clinical variables, a heterogeneity of 0% was obtained, with a not significant mean difference 21.

### Directions for future research

Future work involving self-management evidence-based practices in nursing care are necessary in order to achieve better control of risk factors and negative health outcomes. It is also necessary to enroll families in healthy behaviors for outstanding individual goals. It means that interventions should not only focus on individual care, also on patient families and their environment.

### Implications for clinical practice

To achieve healthy behaviors, the family must be involved, that is, interventions should not focus only on individuals, but also on their families and environment. The results of this study can redirect evidence-based practices in nursing care to achieve better control of risk factors and negative health outcomes.

### Limitations

One of the limitations of the present systematic review and meta-analysis is related to the aforementioned results of heterogeneity, however this may be partially explained by differences in population, intervention, or outcome measurement. Thus, the performance of the subgroup analysis also makes it possible to assess the variability of the results of the included studies.

This review suggests that there are no statistically significant differences between self-management interventions and the usual care given to patients with Acute Coronary Syndrome Compliance with Pharmacological Treatment, lifestyle changes and Changes in Clinical Variables. The small number of studies included in the meta-analysis and their heterogeneity do not allow for the extraction of robust conclusions, hence it is difficult to conclude the effect of self-management interventions in ACS patients ♦
